# To Medical Students

**Published:** 1888-09

**Authors:** 


**To Medical Students.**—The Southern Medical College, Atlanta, Georgia, is now regarded as among the very best Institutions in the United States. The College building is commodious and well arranged. The Chairs are well and ably filled. The curriculum is complete. The Dissecting-room is large and well-fitted up for the purpose. There is a Hospital connected with the College, and every facility exists for imparting a thorough medical education. The Annual Catalogue is now being distributed. Parties who desire a Catalogue should address, W. P. NICOLSON, M.D., DEAN,
Atlanta, Ga.

**Cascara Sagrada.**—This drug seems to be growing in popularity. Recent observations indicate that it has specific virtues for the relief of rheumatism. The Cascara Cordial, as prepared by Parke, Davis & Co., is especially recommended. Its adaptation to the treatment of habitual constipation, as a vehicle for nauseous medicines, and to dispel the effects of alcohol and opium abuse, are now generally conceded.

